# Occupational risk factors for idiopathic pulmonary fibrosis in Southern Europe: a case-control study

**DOI:** 10.1186/s12890-018-0644-2

**Published:** 2018-05-21

**Authors:** Giulia Paolocci, Ilenia Folletti, Kjell Torén, Magnus Ekström, Marco Dell’Omo, Giacomo Muzi, Nicola Murgia

**Affiliations:** 1 Section of Occupational Medicine, Respiratory Diseases and Toxicology, University of Perugia, Piazzale Gambuli, 06100 Perugia, Italy; 2 Department of Occupational and Environmental Medicine, Sahlgrenska University Hospital, Göteborg, Sweden

**Keywords:** Occupational, Workers, UIP, Pulmonary fibrosis

## Abstract

**Background:**

Idiopathic pulmonary fibrosis (IPF) is a chronic, progressive fibrosing interstitial pneumonia of unknown cause associated with the histopathologic and/or radiologic pattern of usual interstitial pneumonia (UIP). Occupational risk factors have been proposed to be associated with UIP. The aim of this case-control study is to evaluate the relationship between UIP pattern and occupational exposure in Southern Europe.

**Methods:**

Sixty nine cases with a UIP radiological pattern at CT-scan were selected from a clinical database of the University Hospital of Perugia, Umbria, between January 2010 and December 2013. Controls (*n* = 277) not reporting doctor diagnosed pulmonary fibrosis, were ascertained casually among general population from the same catching area of cases. Data were collected by a questionnaire used previously in a similar study. Logistic regression models, adjusted for gender, age and smoking, were performed to evaluate the association between UIP and occupational exposure.

**Results:**

Farmers, veterinarians and gardeners (OR = 2.73, 95%CI = 1.47–5.10), metallurgical and steel industry workers (OR = 4.80, 95%CI = 1.50–15.33) were occupations associated with UIP. Metal dust and fumes and organic dust were risk factors for UIP. Increasing the length of occupational exposure in jobs at risk of pulmonary fibrosis, increased the risk of having UIP.

**Conclusions:**

This case control study confirm partially the results from previous similar studies. Some discrepancies could be explained by the different geographical origins of the population under study, reflecting also different occupational exposures.

**Electronic supplementary material:**

The online version of this article (10.1186/s12890-018-0644-2) contains supplementary material, which is available to authorized users.

## Background

Idiopathic pulmonary fibrosis (IPF) is a chronic, progressive, fibrosing interstitial lung disease of unknown cause with a high risk of rapid progression and mortality, the median survival after diagnosis is approximately 2 years [1].

The diagnosis of IPF requires exclusion of other known causes of idiophatic lung diseases, the presence of a usual interstitial pneumonia (UIP) pattern on high resolution CT-scan (HRCT) in patients not subjected to surgical lung biopsy (SLB) and specific combinations of HRCT and SLB patterns in patients subjected to SLB [2].

While several key cellular and molecular events have been identified in the pathogenesis of IPF, such as age-associated telomerase dysfunction [3], incidence of IPF has increased in the last years with regional differences suggesting the pathogenic role of various environmental and occupational exposures [4].

Mechanisms governing the relationship between smoking, gender, occupational exposure and the development of severe pulmonary fibrosis are unknown but likely involve complex interactions between different environmental factors in genetically predisposed individuals [5].

In similar studies made in America [6], Northern Europe [7] and Japan [8] occupational risk factors, including metal, stone and wood dust have been linked to higher risk of developing pulmonary fibrosis, but to date, the consistency of epidemiological evidence is suggestive but not sufficient to infer a causal relationship between several environmental exposure and IPF.

The aim of this study is to evaluate the relationship between UIP pattern and occupational exposure in Southern Europe.

## Methods

The design was a case-control study of the adult population in Perugia, Umbria, Central Italy. 69 consecutive cases were included from the Perugia University Hospital clinical database between January 2010 and December 2013 and a random sample of 308 controls was selected among inhabitants of the same catchment area (Region Umbria). The diagnosis of UIP was based on clinical history, UIP pattern at CT scan and, when available, on open lung biopsy. All cases were consecutively included. Exclusion criteria were: laboratory testing compatible with autoimmune diseases, lung irradiation (radiotherapy), current or previous intake of medication known to cause lung fibrosis, compensation for occupational lung diseases resembling clinically and radiologically UIP (e.g asbestosis). Furthermore, cases with high suspicion, based on medical records and exposure history, of an occupational disease, even if not-compensated, were excluded.

All this information were gathered through the UIP patients medical records available at the Section of Occupational Medicine, Respiratory Diseases and Toxicology of the University Hospital of Perugia. The study protocol was approved by the Regional Ethical Committee (CEAS UMBRIA) and all the participants gave their informed verbal consent to the study. Due to the specific design of the study (telephone questionnaire), the informed consent was verbal. To approve the study, the ethical committee (CEAS UMBRIA) required the presence of a witness during questionnaire administration.

### Questionnaire and definitions

Data were collected through an extensive telephonic questionnaire with items about occupations, duration of this occupations, specific self-reported occupational exposure, smoking habits and diagnosis of pulmonary fibrosis with information on atopy, rhinitis, family autoimmunity and family history of pulmonary fibrosis. The questionnaire has been described in detail elsewhere [7]. The response rate was 100% among cases and 90% among controls. Thus, the final number of controls enrolled in the study was 277.

Occupational exposure was ascertained by the answers to the self-reported questions. Self-reported exposures were lumped in 5 larger categories, metal dust and fumes, mineral dust, organic dust, vapour gas and non-metal fumes, environmental tobacco smoke. To be classified as exposed, a subject had to report an occupation or an exposure lasting for at least 5 years and for cases, starting 5 years or more before the diagnosis of IPF, based on UIP pattern at CT scan.

Another measure of exposure was based on self-reported occupations: job titles were grouped into 12 job categories and of these, 5 have been considered at risk to cause UIP. The selection of the 5 job categories at risk to develop UIP was made through a review of the literature (Additional file 1). The search was conducted in the PubMed electronic database using the following search phrase: “Idiopathic pulmonary fibrosis” or “usual interstitial pneumonia” AND (occupation* OR work* OR environment) considering only review articles, in English language and human studies*.* Therefore, of 68 studies, 11 were selected and based on these 11 review articles, construction, wood, metallurgical and steel and chemical workers, as well as farmers, veterinarians (vets) and gardeners were considered professions at risk to generate the disease.

### Statistical analysis

Significance of differences in prevalence were calculated by Chi-square test, and Fisher’s exact test, when appropriate. Significance of differences in means were calculated using t-test. To evaluate different risk factors, logistic regression models have been built for occupation at risk of UIP and for self-reported exposure to occupational hazards, adjusting for gender, age, and smoking (ever and never). Specific models stratifying for gender and adjusting for age and smoking were also build. Associations were expressed as OR with 95% CI, statistical significance was defined as a double-sided *p* < 0.05. Only exposure categories with five or more exposed cases were considered in the final analysis. Statistical analyses were performed using SPSS statistical software, version 20.0 (SPSS, IBM Corporation, New York, NY, USA).

## Results

The study comprises 69 consecutive patients with UIP radiological pattern and 277 controls. The mean age was 75 years for cases and 71 for controls, 72.5% of cases and 54.2% of controls were males. Cases and controls had the same smoking exposure. No differences were found in distribution of family history pulmonary fibrosis as well as in atopy and rhinitis history between the two groups. Baseline characteristics are shown in Table 1.

**Table 1:** Characteristics of the population

	UIP cases (*n* = 69)	Controls (*n* = 277)	p
Males, n (%)	50 (72.5)	150 (54. 2)	< 0.01
Age, mean ± SD	75 (± 14)	71 (± 13)	0.02
Smoking			
No smokers	27 (39.1)	110 (39.7)	NS
Eversmokers	42 (60.9)	167 (60.3)	
Atopy, n (%)	5 (7.2)	25 (9.0)	NS
Rhinitis, n (%)	22 (31.9)	75 (27.1)	NS
Family history of pulmonary fibrosis, n (%)	3 (4.5)	3 (1.1)	0.09
Family autoimmunity, n (%)	7 (10.1)	33 (11.9)	NS
Work duration (years), mean ± SD	38.6 (± 10.6)	35.4 (± 10.5)	0.02
Job title			
Legislators, entrepreneurs, scientific and intellectual professions	4 (5.8)	21 (7.6)	NS
Technical, social and health professions	9 (13)	62 (22.4)	NS
Office workers	11 (15.9)	48 (17.3)	NS
Dealers and service workers	11 (15.9)	63 (22.7)	NS
Drivers or operatives of vehicles and installations	6 (8.7)	42 (15.2)	NS
Construction workers	14(20.3)	26 (9.4)	0.02
Food industry workers	5 (7.2)	30 (10.8)	NS
Textile industry workers	9 (13)	36 (13)	NS
Wood industry workers	6 (8.7)	15 (5.4)	NS
Metallurgical and steel industry	8 (11.6)	6 (2.2)	0.02
Farmers, vets and gardeners	30 (43.5)	51 (18.4)	< 0.01
Chemical industry	2 (2.9)	0 (0)	0.04

Subjects with any occupational exposure to agents known to cause UIP had an increased risk for UIP (OR 4.1 95% CI 2.3–7.55) (Table 2).

**Table 2:** Job at risk for UIP in the whole sample, in men and women, adjusted for age and smoking

	All^a^	Men	Women
Exposure	OR (95% CI)	OR (95% CI)	OR (95% CI)
None	1	1	1
Any job at risk of UIP	*4.14 (2.27–7.53)*	*4.40 (2.13–9.05)*	*3.91 (1.18–12.86)*
Costruction workers	1.31 (0.58–2.98)	1.37 (0.60–3.12)	–
Wood industry workers	1.36 (0.46–3.97)	1.35 (0.46–3.95)	–
Metallurgical and steel industry	*4.80 (1.50–15.33)*	*4.76 (1.50–15.15)*	–
Farmers, vets and gardeners	*2.73 (1.47–5.10)*	*2.42 (1.14–5.11)*	*3.91 (1.18–12.86)*

Significant risk estimates in italic

^a^the whole sample also adjusted for gender

To have worked in a metallurgical and steel industry (OR 4.8 95% CI 1.5–15.3) and to be a farmer, vet or gardener (OR 2.7 95% CI 1.5–5.1) increased the risk of UIP (Table 2). There was an association between a longer occupational exposure in any job at risk of UIP and the risk of UIP (Table 3).

**Table 3:** Effect of the length of the occupation in a job at risk of UIP and the risk of UIP, adjusted for sex, age and smoking

Exposure	OR	95% CI
None	1	
1–9 years	*3.32*	*1.06–10.33*
10–19 years	*3.41*	*1.30–8.95*
≥ 20 years	*5.01*	*2.55–9.84*

Significant risk estimates in italic

The self-reported exposure confirmed the findings obtained from the job title analysis underlying an excess of risk if the subject reported the exposure to metal dust and fumes or organic dust. In this latter analysis a self-reported exposure to environmental tobacco smoke doubled the risk of UIP (Table 4).

**Table 4:** Self-reported exposure and risk for UIP, adjusted for age, gender and smoking

Occupational exposure	Cases n	Controls n	OR (95%CI)
Metal Dust or metal fumes	9	9	*3.8 (1.2–12.2)*
Mineral Dust	23	39	1.7 (0.8–3.6)
Organic Dust	37	104	*2.4 (1.3–4.3)*
Vapours, gas, fumes	25	85	0.9 (0.5–1.7)
Environmental tobacco smoke at work	43	116	*2.2 (1.2–4.0)*

Significant risk estimates in italic

When the analysis was made considering also those who have reported shorter exposure (< 5 years) the results did not change (data not shown).

## Discussion

The main finding of this study was the increased risk of UIP among those with a job known to be at risk of UIP. In detail, farmers, veterinarians, gardeners, metal and steel workers were at risk of UIP. Self-reported exposure to metal dust, fumes and organic dust was confirmed as a risk factor for UIP. This association provided support for the hypothesis that pulmonary fibrosis may be a heterogeneous disorder caused by a number of environmental and occupational exposures. The same risk factors have been found in observational studies from different countries [9]. Agricultural workers, veterinarians and gardeners are exposed to organic dust and aerosolized particulates from a variety of sources, including feed grains, bedding and cattle fecal material. The contribution of this exposure to the pathogenesis of lung fibrosis is not completely clear, but, even if all the cases were negative for common serum precipitins, the presence of end-stage unrecognized chronic hypersensitivity pneumonitis can not be excluded. In fact, chronic hypersensitivity pneumonitis could have an UIP radiological pattern [10] and can be confused with idiopathic pulmonary fibrosis, also by experienced physicians [11]. Moreover, veterinarians are usually less exposed to organic dust than agricultural workers and gardener, in this study the contribution of this job title (veterinarians) to the risk of UIP was quite low, since we had just one controls doing this job and his exclusion from the study did not change significantly the results.

The association between metal exposure and lung fibrosis is demonstrated in original studies [6] and review [12]. Metal-related lung fibrosis could be explained by the transition of alveolar cells to mesenchymal cells, caused by reactive oxygen species [13], where some metals are well established inducers of reactive oxygen species [14]. However, asbestos exposure in steel and metallurgy, as well in construction industry, can not be excluded [15], especially in those working in steel mills before ‘90s.

In a previous similar study an association between severe pulmonary fibrosis and wood exposure was found; in this study wood dust exposure did not affect significantly the risk of IPF. This finding could be explained by the different geographical origins of the population considered, reflecting also different economic and productive situation and thus occupational exposures. As a matter of fact, in the present study, the number of subjects exposed to wood dust was rather low compared to the previous study [7].

The association with occupational exposure was time-dependent: the risk of IPF increases with the duration of exposure. The finding of a dose-response relationship between years of potential exposure and risk of disease confirmed previous findings [16, 17]. The exposure assessment in this study was not based only on self-reported exposure, easily prone to recall bias by IPF patients, but also on a job exposure matrix, grounded on previous studies merged in reviews. The reason to consider just reviews was to have a more homogeneous source of information. Anyway, to avoid misclassification, an a posteriori literature search among original articles was performed, confirming the same results obtained with reviews- restricted search. Anyway, we can not exclude exposure misclassification, beside the possibile asbestos exposure in steel and metallurgy, carpenters, classified as wood workers could have been also exposed to asbestos, as well many construction workers.

In this study, we do not perform matching and gender distribution was not similar among cases and controls. Nevertheless, stratified analysis for gender led to the same results in men and women (data not shown), even if women did not report to work or have worked in some occupations (construction, wood and metal).

In the International Consensus Statement regarding the diagnosis and treatment of IPF, Costabel et al. cited cigarette smoking as a potential risk factor for IPF [18] and it have shown in previous studies a dose-related association with increased risk of severe pulmonary fibrosis [19]. Our findings do not support this association, maybe because ever-smoking prevalence, despite the recent introduction of restrictions, is still rather high in Southern Europe, compared to other western countries from where the majority of previous studies were coming. The high prevalence of smokers could be one of the reason of a very high prevalence of self-reported exposure to environmental tobacco smoke in this study among UIP patients (62.3%), although a recall bias or same artifact in the statistical analysis cannot be excluded.

One of the strengths of the present study is the case definition, which includes cases with a medical diagnosis of UIP based on medical records with rather strict exclusion criteria, lung CT-scan UIP radiological pattern and, when available, histological data. In previous studies, case definition by medication registry (e.g. liquid oxygen therapy registry) could yield to misclassification and survivor bias [20]. Moreover, the recruitment of controls from the general population of the same catchment area should avoid bias related to the presence of shared risk factors (smoking, occupation) in an hospital-based case-control design. Not having imaging from control subjects could lead to a classification bias, if some of the controls has a not yet diagnosed pulmonary fibrosis; however, the very low prevalence of UIP in the general population (8.2 cases/100000) [21] could reduce the risk of this bias.

The reason to exclude exposure in the five years before the diagnosis is based on the nature of IPF which is chronic and progressive and a minimum lag time of five years was considered reasonable, based on previous studies dealing with occupational exposures to a known fibrogenic agent, such as silica, and the occurrence of non-malignant fibrotic lung diseases, such as silicosis [22]. However, in a case-control study design, the criterion of temporality, with exposure preceding the onset of UIP, cannot be established safely, because exposure and disease are measured, unlike cohort study, simultaneously [23] and UIP, as other chronic diseases, could also have been diagnosed later than the real onset.

A possible limitation of this study is the low number of cases, but other previous [23] and more recent studies, even if multicenter, have considered similar numbers [24].

Lack of matching for gender and age could be seen as another potential limitation, but the homogeneity of the sample, recruited in the same area and the results after adjustment for age and gender in the whole model and stratification for gender, could contribute to reduce the burden of this limitation on risk estimates. Moreover, it is not clear whether gender is a strong confounder (e.g. for hormone-related issues or for some protective effect of pregnancy on UIP) or an intermediate factor because the exposure to some occupational or environmental risk factor for UIP is more likely to occur in men. Therefore, to avoid underestimation, we decide to not match for gender.

Another possible drawback of a case-control design is the risk of recall bias regarding the exposure assessment. However, in this study, the use of a job exposure matrix would reduce significantly the risk of recall bias in cases and controls.

Another limitation, difficult to overcome given this study design, is the change in exposure intensity over years [25], it is plausible that younger participants underwent to a lighter exposure than the older ones. A larger number of cases could have allowed a stratified analysis for age, which could take into account this aspect.

Finally, some important risk factor for UIP, such as acid microaspiration in gastroesophageal reflux (GER), have not been taken into account in this study. However, GER have been found frequently in UIP patients but its role in causation is still rather unclear, furthermore, diagnostic tools to evaluate the occurrence of GER prior the diagnosis of UIP are still lacking [26].

## Conclusions

Despite some potential limitations, this study confirm data from previous epidemiological studies and overall stress the importance of occupational and environmental factors in UIP pathogenesis. A better understanding of the risk factors for UIP is still needed to prevent its occurrence and, in order to assess the real impact of occupational exposure, further studies are required in larger population samples.

## Additional file

Additional file 1: Flow chart showing the selection process of the literature used to highlight occupational exposures considered at risk of IP. (DOCX 68 kb)

## Abbreviations

GER: Gastro esophageal reflux
HRCT: High resolution CT-scan
IPF: Idiopathic pulmonary fibrosis
SLB: Surgical lung biopsy
UIP: Usual interstitial pneumonia
