# Supplementary material for: Occupational risk factors for idiopathic pulmonary fibrosis in Southern Europe: a case-control study
Source: BMC Pulm Med. 2018 May 21;18:75. doi: 10.1186/s12890-018-0644-2 (PMC5963078; doi:10.1186/s12890-018-0644-2)
Supplement: Supplementary file 1 — Flow chart showing the selection process of the literature used to highlight occupational exposures considered at risk of IP. (DOCX 68 kb) [file 12890_2018_644_MOESM1_ESM.docx]

**Figure 1 : Flow chart showing the selection process of the literature used to highlight occupational exposures considered at risk of IP**

PUBMED DATABASE: “Idiopathic pulmonary fibrosis” or “usual interstitial pneumonia” AND (occupation* OR work* OR environment)

Filters: review, English language, human studies

43 studies were excluded by title

68 studies were identified

14 studies were excluded by abstract

25 abstracts were analyzed

*11 studies were reviewed

**Construction workers**

**Wood industry workers**

**Metallurgical and steel industry** workers = workers at risk of IPF

**Chemical workers**

**Farmers, vets and gardeners**

***Articles selected**

1. [**Pathogenesis of idiopathic pulmonary fibrosis and its clinical implications.**](http://www.ncbi.nlm.nih.gov/pubmed/24953006) Spagnolo P, Rossi G, Cavazza A. Expert Rev Clin Immunol. 2014 Aug;10(8):1005-17. doi: 10.1586/1744666X.2014.917050. Epub 2014 Jun 23. Review.

2. **Workshop on idiopathic pulmonary fibrosis in older adults.** [Castriotta RJ](http://www.ncbi.nlm.nih.gov/pubmed/?term=Castriotta%20RJ%5BAuthor%5D&cauthor=true&cauthor_uid=20822991), [Eldadah BA](http://www.ncbi.nlm.nih.gov/pubmed/?term=Eldadah%20BA%5BAuthor%5D&cauthor=true&cauthor_uid=20822991), [Foster WM](http://www.ncbi.nlm.nih.gov/pubmed/?term=Foster%20WM%5BAuthor%5D&cauthor=true&cauthor_uid=20822991), [Halter JB](http://www.ncbi.nlm.nih.gov/pubmed/?term=Halter%20JB%5BAuthor%5D&cauthor=true&cauthor_uid=20822991), [Hazzard WR](http://www.ncbi.nlm.nih.gov/pubmed/?term=Hazzard%20WR%5BAuthor%5D&cauthor=true&cauthor_uid=20822991), [Kiley JP](http://www.ncbi.nlm.nih.gov/pubmed/?term=Kiley%20JP%5BAuthor%5D&cauthor=true&cauthor_uid=20822991), [King TE Jr](http://www.ncbi.nlm.nih.gov/pubmed/?term=King%20TE%20Jr%5BAuthor%5D&cauthor=true&cauthor_uid=20822991), [Horne FM](http://www.ncbi.nlm.nih.gov/pubmed/?term=Horne%20FM%5BAuthor%5D&cauthor=true&cauthor_uid=20822991), [Nayfield SG](http://www.ncbi.nlm.nih.gov/pubmed/?term=Nayfield%20SG%5BAuthor%5D&cauthor=true&cauthor_uid=20822991), [Reynolds HY](http://www.ncbi.nlm.nih.gov/pubmed/?term=Reynolds%20HY%5BAuthor%5D&cauthor=true&cauthor_uid=20822991), [Schmader KE](http://www.ncbi.nlm.nih.gov/pubmed/?term=Schmader%20KE%5BAuthor%5D&cauthor=true&cauthor_uid=20822991), [Toews GB](http://www.ncbi.nlm.nih.gov/pubmed/?term=Toews%20GB%5BAuthor%5D&cauthor=true&cauthor_uid=20822991), [High KP](http://www.ncbi.nlm.nih.gov/pubmed/?term=High%20KP%5BAuthor%5D&cauthor=true&cauthor_uid=20822991). [Chest.](http://www.ncbi.nlm.nih.gov/pubmed/?term=workshop+on+idiopathic+pulmonary+fibrosis%2C+castriotta) 2010 Sep;138(3):693-703. doi: 10.1378/chest.09-3006.

3. **Geoepidemiology of COPD and idiopathic pulmonary fibrosis.** [Zeki AA](http://www.ncbi.nlm.nih.gov/pubmed/?term=Zeki%20AA%5BAuthor%5D&cauthor=true&cauthor_uid=20018478), [Schivo M](http://www.ncbi.nlm.nih.gov/pubmed/?term=Schivo%20M%5BAuthor%5D&cauthor=true&cauthor_uid=20018478), [Chan AL](http://www.ncbi.nlm.nih.gov/pubmed/?term=Chan%20AL%5BAuthor%5D&cauthor=true&cauthor_uid=20018478), [Hardin KA](http://www.ncbi.nlm.nih.gov/pubmed/?term=Hardin%20KA%5BAuthor%5D&cauthor=true&cauthor_uid=20018478), [Kenyon NJ](http://www.ncbi.nlm.nih.gov/pubmed/?term=Kenyon%20NJ%5BAuthor%5D&cauthor=true&cauthor_uid=20018478), [Albertson TE](http://www.ncbi.nlm.nih.gov/pubmed/?term=Albertson%20TE%5BAuthor%5D&cauthor=true&cauthor_uid=20018478), [Rosenquist GL](http://www.ncbi.nlm.nih.gov/pubmed/?term=Rosenquist%20GL%5BAuthor%5D&cauthor=true&cauthor_uid=20018478), [Louie S](http://www.ncbi.nlm.nih.gov/pubmed/?term=Louie%20S%5BAuthor%5D&cauthor=true&cauthor_uid=20018478). [J Autoimmun.](http://www.ncbi.nlm.nih.gov/pubmed/?term=geoepidemiology+of+COPD+and+idiopathic+pulmonary+fibrosis%2C+ZEki) 2010 May;34(3):J327-38. doi: 10.1016/j.jaut.2009.11.004. Epub 2009 Dec 16.

4. **Exposures and idiopathic lung disease.** [Taskar V](http://www.ncbi.nlm.nih.gov/pubmed/?term=Taskar%20V%5BAuthor%5D&cauthor=true&cauthor_uid=19221965)^1^, [Coultas D](http://www.ncbi.nlm.nih.gov/pubmed/?term=Coultas%20D%5BAuthor%5D&cauthor=true&cauthor_uid=19221965). [Semin Respir Crit Care Med.](http://www.ncbi.nlm.nih.gov/pubmed/19221965) 2008 Dec;29(6):670-9. doi: 10.1055/s-0028-1101277. Epub 2009 Feb 16.

5. [**Clinical year in review III: Idiopathic pulmonary fibrosis, occupational medicine, and lung transplantation.**](http://www.ncbi.nlm.nih.gov/pubmed/18757312) Wilkes DS. Proc Am Thorac Soc. 2008 Sep 15;5(7):751-4. doi: 10.1513/pats.200806-051TT. Review.

6. [**Idiopathic pulmonary fibrosis: evolving concepts.**](http://www.ncbi.nlm.nih.gov/pubmed/24867394) Ryu JH, Moua T, Daniels CE, Hartman TE, Yi ES, Utz JP, Limper AH. Mayo Clin Proc. 2014 Aug;89(8):1130-42. doi: 10.1016/j.mayocp.2014.03.016. Epub 2014 May 24. Review.

7. [**Host-environment interactions in pulmonary fibrosis.**](http://www.ncbi.nlm.nih.gov/pubmed/17195134) Garantziotis S, Schwartz DA. Semin Respir Crit Care Med. 2006 Dec;27(6):574-80. Review.

8. **Is idiopathic pulmonary fibrosis an environmental disease?** [Taskar VS](http://www.ncbi.nlm.nih.gov/pubmed/?term=Taskar%20VS%5BAuthor%5D&cauthor=true&cauthor_uid=16738192), [Coultas DB](http://www.ncbi.nlm.nih.gov/pubmed/?term=Coultas%20DB%5BAuthor%5D&cauthor=true&cauthor_uid=16738192). [Proc Am Thorac Soc.](http://www.ncbi.nlm.nih.gov/pubmed/16738192) 2006 Jun;3(4):293-8.

9. [**Occupational dust exposure and the aetiology of cryptogenic fibrosing alveolitis.**](http://www.ncbi.nlm.nih.gov/pubmed/11816818) Hubbard R. Eur Respir J Suppl. 2001 Sep;32:119s-121s. Review.

10. [**Recent advances in particulate-induced pulmonary fibrosis; for the application of possible strategy experimentally and clinically.**](http://www.ncbi.nlm.nih.gov/pubmed/11465077) Kim KA, Park CY, Lim Y, Lee KH. Curr Drug Targets. 2000 Nov;1(3):297-307. Review

11. [**Smoking-related interstitial lung diseases.**](http://www.ncbi.nlm.nih.gov/pubmed/10958232) Nagai S, Hoshino Y, Hayashi M, Ito I. Curr Opin Pulm Med. 2000 Sep;6(5):415-9. Review.
